# Clinical characteristics and risk factors of severe COVID-19 in hospitalized neonates with omicron variant infection: a retrospective study

**DOI:** 10.1186/s13052-024-01751-5

**Published:** 2024-09-16

**Authors:** Huijing Wei, Fu Wei, Xiaokang Peng, Pan Liu, Li Tang, Yishan Liu, Shan Liao, Yajing Bo, Yuzhen Zhao, Ruina Li, Xiaoguai Liu, Fanpu Ji

**Affiliations:** 1 Department of infectious Diseases, Xi’an Jiaotong University Affiliated Children’s Hospital, No. 69 Xi Ju Yuan Alley, Xi’an, 710003 Shaanxi China; 2 Department of Critical Care Medicine, Xi’an Fourth Hospital, Xi’an People’s Hospital, Xi’an City, Shaanxi Province China; 3 Department of infectious Diseases, The Second Affiliated Hospital Xi’an Jiaotong University, No.157 Xi Wu Road, Xi’an, Shaanxi Province 710004 China; 4 Department of Clinical Laboratory, The Second Affiliated Hospital of Xi’an Jiaotong University, Xi’an, China; 5 National & Local Joint Engineering Research Center of Biodiagnosis and Biotherapy, the Second Affiliated Hospital of Xi’an Jiaotong University, Xi’an, Shaanxi China; 6 Shaanxi Provincial Clinical Medical Research Center of Infectious Diseases, Xi’an, Shaanxi China; 7 Key Laboratory of Surgical Critical Care and Life Support (Xi’an Jiaotong University), Ministry of Education, Xi’an, Shaanxi China; 8 Key Laboratory of Environment and Genes Related to Diseases, Xi’an Jiaotong University, Ministry of Education of China, Xi’an, Shaanxi China

**Keywords:** Neonates, SARS-CoV-2, Omicron variant, Severe COVID-19, Risk factors

## Abstract

**Background:**

Reports on coronavirus disease 2019 (COVID-19) in neonates are limited, especially in patients infected with severe acute respiratory syndrome coronavirus 2 (SARS-COV-2) Omicron variant. This study aims to analyze the clinical characteristics and identify risk factors associated with severe COVID-19 in neonates infected with Omicron variant.

**Methods:**

The study population was represented by neonates with COVID-19, who were admitted to The Affiliated Children’s Hospital of Xi’an Jiaotong University in northwest China, from December 10, 2022 to January 20, 2023. Chinese Center for Disease Control and Prevention (CDC) announced that all local COVID-19 cases were infected with Omicron variant during the study period. Clinical and laboratory data were collected retrospectively. We used logistic regression analysis to investigate the risk factors for severe COVID-19, and derived odds ratios (ORs) and the corresponding 95% confidence intervals (CIs).

**Results:**

A total of 108 neonates, with median age of 18.1 days (interquartile range 9.4–23.0), were affected by COVID-19, of whom 84 had a mild disease, while 24 a severe one (22.2%). Of them, 6.5% were premature. No deaths were observed in the study population. The most common clinical manifestations were fever (88.9%) and cough (55.6%), with 5 cases (4.6%) complicated by pneumonia. 4 cases (3.7%) received respiratory support, including 2 cases of high-flow oxygen and 2 cases of continuous positive airway pressure. Gestational age at birth (OR: 0.615; 95% CI: 0.393–0.961), neutrophil count (NEU) (OR:0.576; 95% CI : 0.344–0.962) and lymphocyte count **(**LYM) (OR: 0.159; 95% CI: 0.063–0.401) were independent risk factors for severe COVID-19. The combination of NEU and LYM had the largest receiver operating characteristic area under the curve [0.912 (95% CI:0.830–0.993)] for identifying severe COVID-19, with a sensitivity of 0.833 and a specificity of 0.917.

**Conclusions:**

The general presentations and outcomes of neonatal COVID-19 caused by Omicron variant were not severe, and very few patients required respiratory support. The simultaneous decrease in NEU and LYM can be used to identify severe infection.

## Background

The novel severe acute respiratory syndrome coronavirus 2 (SARS-CoV-2) causes coronavirus disease 2019 (COVID-19), which led to a global pandemic since early 2020. As of April 19, 2023, the World Health Organization has reported over 760 million diagnoses of COVID-19, resulting in over 6 million deaths globally. Data from the Centers for Disease Control and Prevention in the United States show that children under 16 years old account for 14.5% of all cases of COVID-19, with a mortality of 0.1% in different age groups [1]. COVID-19 remains one of the major infectious diseases threatening global public health.

Compared to adults and older children, the immunological development of neonates is incomplete. Therefore, age may play a role in distinct clinical characteristics and outcomes in patients infected with SARS-CoV-2. The incidence of neonatal COVID-19 is low, ranging from 5.6 to 10 per 10,000 [2, 3]. A multicentre study of COVID-19 in children and adolescents in Europe showed that the proportion of infants under 1 month old was 7%, which was the lowest of all the age groups [4]. At present, studies on neonatal COVID-19 primarily focus on special cases [5] and on neonates born to mothers infected with SARS-CoV-2 [6–8]. Thus, more studies on the clinical features and the factors associated with severe neonatal COVID-19 are needed [2, 3, 9]. 

SARS-CoV-2 is constantly mutating, and the existing variants have caused waves during the COVID-19 pandemic at different times and regions. On November 26, 2021, the World Health Organization announced that the Omicron variant was displaying stronger transmissibility and immune escape, which led increased rates of COVID-19 [10, 11]. According to data announced by the Chinese Center for Disease Control and Prevention, an increase in SARS-CoV-2 positive cases in various provinces of China has been observed since December 9, 2022, and inpatients have continued to decline after reaching the peak on January 5, 2023. From December 1, 2022 to April 20, 2023, the Omicron variant was the predominant strain of SARS-CoV-2 in China. The Omicron variant is less pathogenic than other previous SARS-CoV-2 variants [12, 13]. However, systematic studies on neonatal COVID-19 caused by the Omicron variant are lacking.

We analysed data from neonatal COVID-19 cases in the largest children’s hospital in northwest China during the Omicron variant epidemic. To the best of our knowledge, this may be the first systematic study of COVID-19 caused by the Omicron variant in neonates, and it is the largest case series of neonatal COVID-19 in China. This study was designed to investigate the clinical and laboratory characteristics of neonates with COVID-19 caused by the Omicron variant and identify risk factors associated with severe illness.

## Methods

### Patients

Neonates diagnosed with COVID-19 were selected as subjects for this study. We recruited patients at The Affiliated Children’s Hospital of Xi’an Jiaotong University, Xi’an city, from December 10, 2022 to January 20, 2023. The inclusion criteria were: (1) Age at diagnosis ≤ 28 days; (2) Positive SARS-CoV-2 polymerase chain reaction test on a respiratory sample; and (3) Admission to the hospital. We excluded patients with asymptomatic infection, nosocomial infection, or incomplete information.

### Data collection and definitions

The clinical data of the subjects were obtained through the electronic medical record system. We collected demographic characteristics, clinical manifestations, laboratory data, imaging findings, treatment measures and prognosis.

Severe COVID-19 was defined as meeting at least two of the following three conditions: (1) Any fever (> 37.5 °C), cough, tachypnoea, apnoea, respiratory distress or recession, supplemental oxygen requirement, vomiting, or diarrhoea; (2) Low white blood cell count (< 5 × 10^3^/µL), low lymphocyte count (LYM) (< 1 × 10^3^/µL), or increased C-reactive protein (> 5 mg/L); and (3) Pneumonia confirmed by chest X-ray or computed tomography scan [2, 3]. The following conditions were also considered as severe COVID-19: a multisystem inflammatory syndrome in neonates (MIS-N), or other serious complications caused by COVID-19 infection, which were unanimously determined by a medical group composed of more than three professional doctors.

Vertical transmission was defined as the mother of the neonate testing positive for SARS-CoV-2 within 14 days before delivery, and the neonate diagnosed with confirmed SARS-CoV-2 infection in the first 12 h after birth [2, 3]. Neonates born at less than 37 weeks of gestational age were defined as premature infants, with early preterm infants born at less than 34 weeks and late preterm infants born between 34 and 36 weeks.

### Statistical analysis

Descriptive statistics were presented as frequencies, proportions, means ± standard deviations (SD) and median with appropriate interquartile range (IQRs). Mann-Whitney *U* test, Pearson’s *χ*^*2*^, Fisher’s exact test and *t* test were used to compare the differences in data between two groups as needed. Risk factors associated with severe COVID-19 were established by univariate and multivariate logistic regression analyses. The receiver operating characteristic curve (ROC) was applied to calculate the sensitivity and specificity of the factors associated with severe disease. The area under the curve (AUC), sensitivity and specificity were used to evaluate the accuracy of the factors. All data were computed in SPSS 26.0, and graphs were created in GraphPad Prism 9.0 (La Jolla, CA, USA). The 2-tailed threshold for significance was set at *P* < 0.05.

## Results

### Study population

**Fig. 1:**
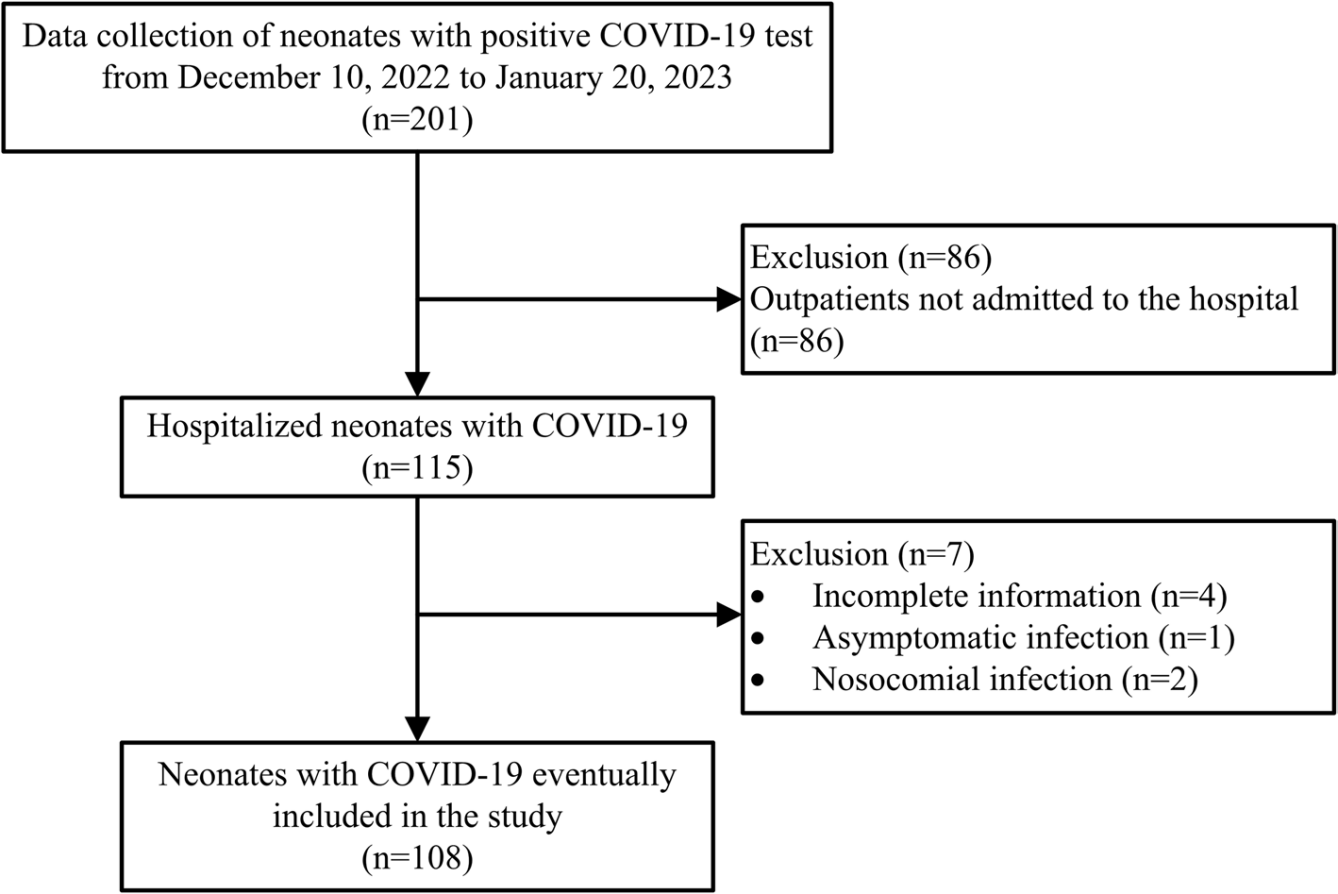
Flow chart of the selection of study population

We selected 108 hospitalized neonates with COVID-19 as the study subjects (Fig. 1), of whom 4 cases were admitted to Neonatal Intensive Care Units and the others were in Neonatology. The proportion of males was 58.3%. The median age at diagnosis was 18.1 days (inter-quartile range 9.4–23.0). 7 cases were premature infants, accounting for 6.5% of our study population. They were classified as late preterm infants with a gestational age of over 34 weeks at birth. Three cases were potentially infected by SARS-CoV-2 through vertical transmission. A definite exposure to a SARS-CoV-2-positive patient was confirmed in 99 cases. In 89.8% of these cases, exposure came from family members including parents, grandparents, and siblings, and 2 cases (1.9%) exposed to other caregivers. It was observed that 71 cases (65.7%) were breastfed after birth, including 33 cases of exclusive breastfeeding and 38 cases of mixed feeding.

### Clinical characteristics and outcomes

**Fig. 2:**
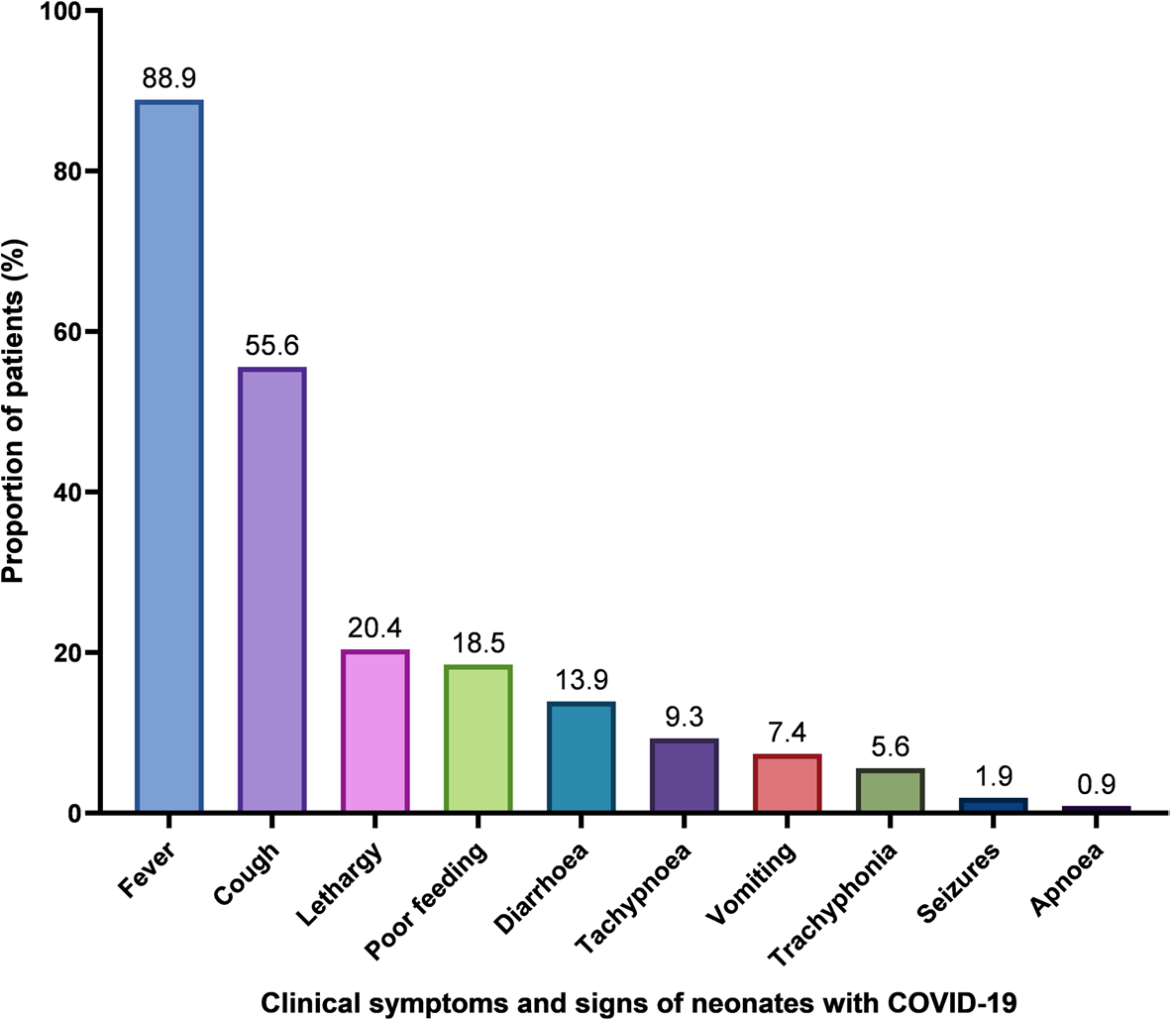
The proportion of clinical symptoms and signs of neonates with COVID-19

Fever and cough were the most common symptoms, accounting for 88.9% and 55.6% of cases, respectively. Other respiratory symptoms included trachyphonia (hoarseness), tachypnoea, and apnoea. There were fewer gastrointestinal and neurological symptoms, such as diarrhea, vomiting and convulsions (Fig. 2).

Two full term neonates (1.9%) experienced seizures and both of them were generalized tonic clonic seizures. Perinatal or congenital causes that may led to seizures, such as hypoxic ischemic encephalopathy, intracranial hemorrhage, hypoglycemia, and intrauterine infections, were not found in either of them. The mothers of these 2 patients had uncomplicated pregnancies and births. While hospitalized, 1 patient experienced an isolated seizure incident, while the other patient experienced several seizures and was suspected of incontinentia pigmenti. The magnetic resonance imaging (MRI) of the patient who experienced several seizures revealed abnormal signal in the left lateral periventricular space, and slightly increased signal in the pallidum bilaterally in T1-weighted sequences. All other sequences and images, including T2-weighted sequences, diffusion-weighted images, and apparent diffusion coefficient maps, showed no abnormalities. MRI findings of the patient who experienced an isolated seizure incident were normal. The electroencephalograms of both patients were mildly abnormal with multifocal spikes, and the cerebrospinal fluid analyses showed no abnormalities.

Among the 108 neonates with COVID-19, 24 cases (22.2%) were diagnosed with severe disease. However, no deaths and MIS-N were recorded. The median length of stay was 4.0 days (IQR 9.4–23.0). There were no significant differences in clinical manifestations between the severe and non-severe groups. However, the white blood cell count, neutrophil count (NEU), and LYM were lower in the severe group (Table 1).

**Table 1:** Demographic, clinical and laboratory characteristics of neonates with non-severe and severe COVID-19

Characteristic	Non-severe group (*n* = 84)	Severe group (*n* = 24)	*P*
Male, *n* (%)	46 (54.8)	17 (70.8)	0.159
Age at admission (d), median (IQR)	15.4 (7.6–22.6)	21.1 (16.4–26.2)	0.023
Weight at admission (kg), mean (SD)	3.7 (0.5)	4.0 (0.7)	0.023
Birth weight (kg), mean (SD)	3.3 (0.4)	3.3 (0.4)	0.981
Gestational age at birth (wk), mean (SD)	39.3 (1.2)	38.8 (1.5)	0.107
Mode of delivery, *n* (%)			0.606
Vaginal	40 (47.6)	10 (41.7)	N/A
Caesarean	44 (52.4)	14 (58.3)	N/A
Feeding pattern, *n* (%)			0.065
Breastfeeding	22 (26.2)	11 (45.8)	N/A
Formula feeding	28 (33.3)	9 (37.5)	N/A
Mixed feeding	34 (40.5)	4 (16.7)	N/A
Contact of patients with COVID-19, *n* (%)			0.312
Family members	77 (91.7)	20 (83.3)	N/A
Other caregivers	1 (1.2)	1 (4.2)	N/A
Unknown	6 (7.1)	3 (12.5)	N/A
Fever, *n* (%)	74 (88.1)	22 (91.7)	0.902
Maximum body temperature in °C, mean (SD)	38.2 (0.4)	38.1 (0.3)	0.190
Fever duration in d, median (IQR)	1.5 (1.0–2.0)	1.0 (1.0–2.0)	0.308
Cough, *n* (%)	49 (58.3)	11 (45.8)	0.277
Cough duration in d, median (IQR)	3.0 (2.0–4.0)	3.0 (2.0–4.5)	0.655
Trachyphonia, *n* (%)	6 (7.1)	0	0.400
Tachypnoea, *n* (%)	7 (8.3)	3 (12.5)	0.824
Apnoea, *n* (%)	0	1 (4.2)	0.222
Vomiting, *n* (%)	4 (4.8)	4 (16.7)	0.128
Diarrhoea, *n* (%)	12 (14.3)	3 (12.5)	1.000
Seizures, *n* (%)	1 (1.2)	1 (4.2)	0.379
Poor feeding, *n* (%)	15 (17.9)	5 (20.8)	0.974
Lethargy, *n* (%)	16 (19.0)	6 (25.0)	0.725
WBC (×10^9^/L), median (IQR)	8.4 (6.6–10.5)	4.5 (4.3–4.9)	< 0.001
NEU (×10^9^/L), median (IQR)	2.6 (1.9–4.2)	1.7 (1.2–2.1)	< 0.001
LYM (×10^9^/L), median (IQR)	3.3 (2.3–4.8)	1.7 (1.1–2.1)	< 0.001
RBC (×10^12^/L), mean (SD)	4.4 (0.9)	4.1 (0.6)	0.061
HB (g/L), mean (SD)	151.1 (30.5)	140.2 (21.7)	0.054
PLT (×10^9^/L), median (IQR)	304.5 (224.5–357.2)	277 (233.5–324.2)	0.402
CRP (mg/L), median (IQR)	0.6 (0.2–2.2)	0.2 (0.2–1.1)	0.085
PCT (ng/mL), median (IQR)	0.1 (0.1–0.2)	0.1 (0.1–0.1)	0.227
TB (µmol/L), median (IQR)	122.4 (57.1–198.9)	193.5 (142.2–223.4)	0.008
DB (µmol/L), median (IQR)	15.8 (8.7–19.9)	16.2 (7.5–19.2)	0.836
ALB (g/L), median (IQR)	37.0 (34.5–39.2)	37.0 (34.6–39.4)	0.825
ALP (U/L), mean (SD)	213.7 (78.1)	233.8 (79.3)	0.269
ALT (U/L), median (IQR)	16.0 (13.0–20.0)	17.5 (12.0–26.2)	0.649
AST (U/L), median (IQR)	36.5 (32.0–46.0)	39.0 (31.0–46.0)	0.574
CK (U/L), median (IQR)	123.0 (91.5–182.8)	106.0 (89.8–131.2)	0.358
CK-MB (U/L), median (IQR)	33.0 (28.5–41.2)	30.0 (21.8–44.5)	0.579
LDH (U/L), median (IQR)	321.5 (270.8–355.8)	273.5 (255.2–315.5)	0.060
Pneumonia on chest X-ray or CT, *n* (%)	0	5 (20.8)	< 0.001
Comorbidities, *n* (%)	83 (98.8)	24 (100)	1.000
Jaundice, *n* (%)	54 (64.3)	21 (87.5)	0.029
Anaemia, *n* (%)	36 (42.9)	13 (54.2)	0.326
Congenital cardiovascular abnormalities, *n* (%)^a^	11 (13.1)	2 (8.3)	0.782
Heart failure, *n* (%)	0	1 (4.2)	0.222
Non-acute intracranial haemorrhage, *n* (%)^b^	4 (4.8)	1 (4.2)	1.000
Hypoxic ischemic encephalopathy, *n* (%)	0	1 (1.2)	0.222
Congenital pulmonary bulla, *n* (%)	1 (1.2)	0	1.000
Respiratory support, *n* (%)	0	4 (16.7)	0.002
High-flow oxygen, *n* (%)	0	2 (8.3)	0.048
Non-invasive ventilation, *n* (%)	0	2 (8.3)	0.048
Invasive ventilation, *n* (%)	0	0	N/A
Length of hospitalisation (d), median (IQR)	4.0 (3.0–5.0)	4.0 (3.0–4.0)	0.779

*ALB* Albumin, *ALT* Alanine aminotransferase, *ALP* Alkaline phosphatase, *AST* Aspartate aminotransferase, *CK* Creatine kinase, *CK-MB* Creatine kinase MB isoenzyme, *COVID-19* Coronavirus disease 2019, *CRP* C-reactive protein, *CT* Computed tomography, *DB* Direct bilirubin, *HB* Haemoglobin, *IQR* Interquartile range, *LDH* Lactate dehydrogenase, *LYM* Lymphocyte count, *N/A* Not applicable, *NEU* Neutrophil count, *PCT* Prothrombin consumption test, *PLT* Platelet, *RBC* Red blood cell count, *SD* Standard deviation, *TB* Total bilirubin, *WBC* White blood cell

^a^Congenital cardiovascular abnormalities included atrial septal defect, interventricular septum defect, pulmonary artery stenosis, and patent ductus arteriosus, with one or more existing simultaneously

^b^Non-acute intracranial haemorrhage was defined as the presence of intracranial haemorrhage confirmed by prenatal cranial ultrasound, computed tomography, or magnetic resonance imaging. The follow-up imaging during hospitalisation showed improvement in absorption

Five cases (4.6%) were complicated with pneumonia, and 4 patients received respiratory support. Two cases required high-flow oxygen (4 L/min) due to decreased peripheral oxygen saturation. The other 2 cases required continuous positive airway pressure due to apnoea in 1 case and respiratory failure in the other one. Most neonates (99.1%) had comorbidity, among which jaundice and anaemia were the most common, followed by congenital cardiovascular abnormalities. Seven patients received antibiotics at admission due to suspicion of sepsis. However, sepsis was ruled out after negative blood cultures. No patients were treated with antiviral drugs, immunoglobulins, or glucocorticoids.

### Factors associated with severe COVID-19

Clinical and laboratory factors associated with severe COVID-19 in neonates were analysed, and factors with a *P* value < 0.2 were included in the univariate logistic regression analysis. The multivariate logistic regression analysis revealed that gestational age at birth [*P* = 0.033, odds ratio (OR) = 0.615, 95% confidence interval (CI) = 0.393–0.961], NEU (*P* = 0.035, OR = 0.576, 95%CI = 0.344–0.962), and LYM (*P* < 0.001, OR = 0.159, 95%CI = 0.063–0.401) were independent risk factors for severe COVID-19 in neonates (Table 2).

**Table 2:** Univariate and multivariate logistic regression analyses of factors associated with severe coronavirus disease 2019 in hospitalised neonates

Factor	Univariate		Multivariate	
	OR (95%CI)	*P*	OR (95%CI)	*P*
Male, *n* (%)	0.498 (0.187–1.327)	0.164	N/A	N/A
Gestational age at birth in wk, mean (SD)	0.747 (0.523–1.068)	0.110	0.615 (0.393–0.961)	0.033
WBC	0.373 (0.233–0.596)	< 0.001	N/A	N/A
NEU	0.667 (0.458–0.971)	0.035	0.576 (0.344–0.962)	0.035
LYM	0.202 (0.094–0.436)	< 0.001	0.159 (0.063–0.401)	< 0.001
PLT	0.988 (0.993–1.002)	0.338	N/A	N/A
hs-CRP	0.776 (0.545–1.104)	0.158	N/A	N/A
PCT	0.628 (0.148–2.677)	0.530	N/A	N/A
TB	1.006 (1.001–1.011)	0.022	N/A	N/A
ALB	0.979 (0.889–1.078)	0.668	N/A	N/A
ALT	1.014 (0.965–1.065)	0.591	N/A	N/A
CK-MB	1.004 (0.996–1.012)	0.287	N/A	N/A
LDH	1.000 (0.996–1.003)	0.896	N/A	N/A

*ALB* Albumin, *ALT* Alanine aminotransferase, *CI* Confidence interval, *CK-MB* Creatine kinase MB isoenzyme, *hs-CRP* high-sensitivity C-reactive protein, *LDH* Lactate dehydrogenase, *LYM* Lymphocyte count, *N/A* Not applicable, *NEU* Neutrophil count, *OR* Odds ratio, *PCT* Prothrombin consumption test, *PLT* Platelet, *SD* Standard deviation, *TB* Total bilirubin, *WBC* White blood cell

**Fig. 3:**
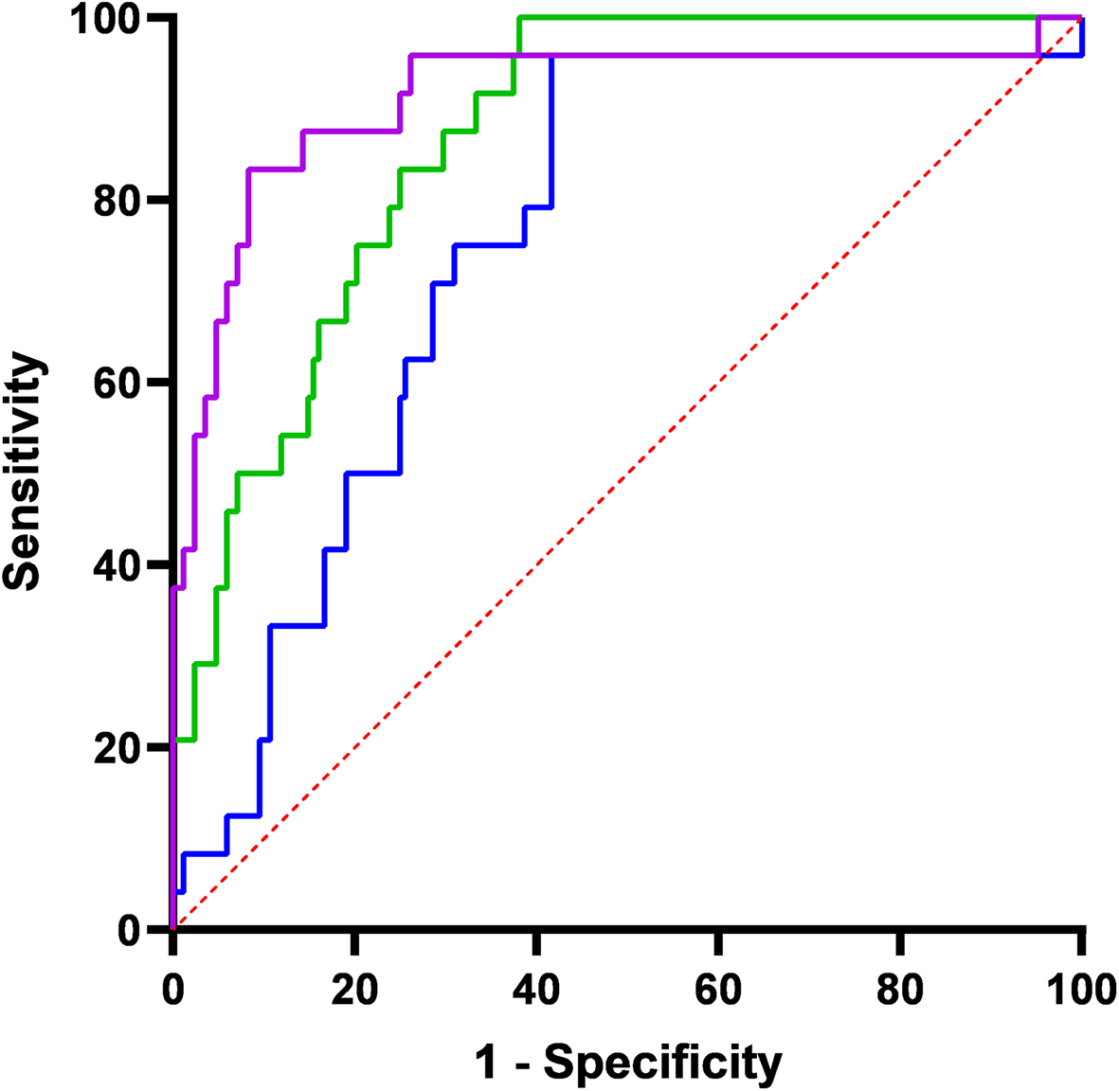
Receiver operating characteristic curves for the independent and combined laboratory parameters to predict severe COVID-19 in neonates. LYM, lymphocyte count; NEU, neutrophil count

We analysed the accuracy of the NEU and LYM laboratory data. The AUC of NEU was 0.750 (95%CI = 0.647–0.854), with a sensitivity of 0.958, a specificity of 0.583, and a cutoff value of 2.390 × 10^9^/L. The AUC of LYM was 0.867 (95%CI = 0.799–0.936), with a sensitivity of 1.000, a specificity of 0.619, and a cutoff value 2.945 × 10^9^/L. The AUC of the combination of NEU and LYM was 0.912 (95%CI = 0.830–0.993), which was higher than the AUC of NEU and LYM separately. The sensitivity and specificity were 0.833 and 0.917, respectively (Fig. 3; Table 3).

**Table 3:** Receiver operating characteristic curve analysis of the independent laboratory factors by multivariate regression analysis for severe coronavirus disease 2019 in neonates

Factor	AUC	95%CI	Sensitivity	Specificity	Cutoff value
NEU	0.750	0.647–0.854	0.958	0.583	2.390
LYM	0.867	0.799–0.936	1.000	0.619	2.945
NEU + LYM	0.912	0.830–0.993	0.833	0.917	N/A

*AUC* Area under the curve, *CI* Confidence interval, *LYM* Lymphocyte count, *NEU* Neutrophil count, *N/A* Not applicable

## Discussion

In this study, we investigated the risk factors associated with severe COVID-19 in neonates admitted to Neonatology and Neonatal Intensive Care Units of The Affiliated Children’s Hospital of Xi’an Jiaotong University, during the Omicron variant epidemic in China. To the best of our knowledge, this is the largest case series on neonatal COVID-19 in China. Among the 108 hospitalised neonates with COVID-19, 22.2% had a severe infection, which was lower than the proportion reported in a previous study (42%) [2]. Only 4 patients (3.7%) required respiratory support, and no patients were treated with invasive ventilation. The proportion of respiratory support was lower than that of other previous reports [2, 3, 9]. These differences may be related to different study populations and weak pathogenicity of the Omicron variant. In addition, the low proportion of premature neonates (6.5%) and the absence of early premature patients in our study may contribute to the differences. No patient was treated with antiviral drugs, immunoglobulins, or glucocorticoids, and there were no deaths, confirming that neonatal COVID-19 typically has a good prognosis [14]. 

Neonatal COVID-19 can affect multiple systems, resulting in respiratory, digestive, or neurological symptoms [15, 16]. These manifestations are similar to those of adults and older children diagnosed with COVID-19. Fever and cough were the most common symptoms of neonatal COVID-19 in our study. Trachyphonia (hoarseness), tachypnoea, apnoea, and pneumonia were among the least common findings. The incidence of respiratory symptoms was lower than previous studies [2, 3, 9]. The main reason for this difference may be that the Omicron variant does not replicate in the upper respiratory tract as robustly as other SARS-CoV-2 variants [12, 17]. There were no significant statistical differences in the various clinical manifestations of neonatal COVID-19 between the severe and non-severe groups, indicating that there were no specific symptoms that could identify a severe infection.

Compared to adults and older children, neonates are more susceptible to infection due to the immature development of their organs, and passive acquisition of antibodies from the mother is a key factor in establishing immunity. Breast milk contains various bioactive factors, such as different types of immunoglobulins, that can promote the development of immune function in neonates and prevent different infectious diseases [18–20]. In this study, 65.7% patients were breastfed after birth, which could provide some protection for the neonates. When a pregnant woman is infected with SARS-CoV-2, the maternal specific immunoglobulin G can be transferred to the foetus through the placenta. This would theoretically provide protection against COVID-19 in neonates. However, the maternal protection is uncertain due to multiple factors such as maternal antibody concentration, placental transfer rate, and time from infection to delivery [21–23]. A previous study showed that most neonates who tested positive for SARS-CoV-2 after birth were asymptomatic [24]. In this study, we were only able to confirm that a few mothers were infected with SARS-CoV-2 during pregnancy. Most mothers of neonates in this study, who received the swab test for virus detection, were not infected with SARS-CoV-2. Therefore, we hypothesize that protection from mothers against SARS-CoV-2 infection was negligible.

Pregnant women receiving the COVID-19 vaccine can have a strong immune response, and the specific antibodies produced after vaccination can be transferred to the foetus or neonate through the placenta or breast milk, providing effective protection for them in the first few months of life [25, 26]. Due to the unknown efficacy and safety of the COVID-19 vaccine at that time, pregnant women were excluded during the clinical trial phase of vaccine development in China. Therefore, all mothers of the neonates in our study were not vaccinated with the COVID-19 vaccine during pregnancy.

The severity of COVID-19 is also influenced by age, and COVID-19 in children is milder than in adults [27, 28]. Excessive immune response and secondary immunological injury can lead to severe COVID-19 in adults. In general, children have stronger immunologic tolerance to COVID-19 that results in a lower immune response and damage [29, 30]. Angiotensin-converting enzyme 2 (ACE2) and transmembrane protein serine 2 (TMPRSS2) are two important receptors for SARS-CoV-2 entry into host cells. Several studies have confirmed that the expression of ACE2 and TMPRSS2 in children is lower than in adults, which may be another potential factor for the decreased severity of the disease in such population [31–33]. The expression of ACE2 and TMPRSS2 in the nasal endothelium of neonates, whether at term or preterm, is lower than that of adults, which may explain the mildness of neonatal COVID-19 due to the Omicron variant [34]. 

The incidence of neurological complications in children with COVID-19 is approximately 7%, and is associated with an increase in disease severity [35]. Neonatal COVID-19 often has no or very few specific neurological symptoms [2, 3, 9]. The mechanisms of neurological complications in COVID-19 patients include the direct invasion of SARS-CoV-2, immune damage mediated by infection, and systemic diseases involving the nervous system [36]. In this study, 2 patients experienced seizures, and the electroencephalograms revealed mild abnormalities, although the role of comorbidities affecting the central nervous system (as for example incontinentia pigmenti suspected in one of them), could not be be excluded.

This study found that gestational age at birth, NEU, and LYM are independent risk factors for severe COVID-19 in neonates. Previous studies have shown that premature birth is one of the risk factors for severe COVID-19 in young children or infants [37, 38]. Premature infants are more susceptible to infection due the immature development of their organs [39]. In addition, the ability of T cells (which play an important role in viral infection) in premature neonates to produce C-X-C motif chemokine ligand 8 is insufficient, which may lead to serious complications and even adverse outcomes after infection [40]. It has been confirmed that intrauterine infection is related to low-birth weight and/or small for gestational age status [41]. In our study, maternal data were not available and only three cases were potentially infected by SARS-CoV-2 through vertical transmission, so it was still unclear whether COVID-19 infection will lead to high risk of intrauterine growth disorders.

The decrease of LYM in peripheral blood is associated with severe COVID-19 in our study, which is consistent with the results of other reports. This may be caused by T cell immune dysfunction [42, 43]. NEU is one of the indicators reflecting inflammation in the body, and its increase often indicates a strong inflammatory response, such as multisystem inflammation syndrome in children [44]. Currently, few reports have shown that COVID-19 in neonates or young infants can lead to a decrease in NEU levels [45]. Although the NEU level was decreased in neonates with severe COVID-19, the mechanism is not yet clear. We hypothesize that the temporary decrease in NEU levels is similar to the decrease in NEU during other viral infections. The decrease in NEU may also be associated with different immune responses in neonates or young infants after infection caused by the Omicron variant.

There were some limitations in this study. First, the study was a retrospective study. All neonates with COVID-19 were from a single centre, which was a children’s hospital located in northwestern China. Therefore, the results may not be applicable to all neonates with COVID-19. Second, the data were collected during a period when the Omicron variant was the only detected SARS-CoV-2 strain in China. Therefore, the results only reflect the clinical characteristics of neonatal COVID-19 caused by such variant and may not be applicable to other SARS-CoV-2 ones. Lastly, maternal data in relation to neonatal COVID-19 were lacking. For example, we were unable to obtain information on specific antibodies against SARS-CoV-2 in serum or breast milk. Further research investigating the protection of maternal antibodies on neonates infected with the Omicron variant is needed.

## Conclusion

We conducted the largest case series of neonatal COVID-19 caused by the Omicron variant in hospitalized infants to determine risk factors associated with severe form of the disease. The general manifestations of COVID-19 in neonates caused by Omicron variant are not severe. The simultaneous decrease in NEU and LYM can be used to identify severe infection in a simple and convenient manner. By conducting routine blood tests at clinics or community hospitals, doctors can identify potentially severely infected newborns and promptly refer them to a specialized hospital.

## Data Availability

The datasets generated during and/or analysed during the current study are available from the corresponding author upon reasonable request.

## Abbreviations

ALT: Alanine aminotransferase
AST: Aspartate aminotransferase
AUC: Area under the curve
BUN: Blood urea nitrogen
CK-MB: Creatine kinase-MB form
COVID-19: Coronavirus disease 2019
MRI: Magnetic resonance imaging
NLR: Neutrophil-to-lymphocyte ratio
OR: Odds ratio
PCT: Procalcitonin
ROC: Receiver operating characteristics
SARS-CoV-2: Severe acute respiratory syndrome coronavirus 2
TB: Total bilirubin
95% CI: 95% confidence interval
